# Apiol-Rich and Caryophyllene-Oxygenated Essential Oils from Amazonian *Piper* Species as Dual-Action Biopesticides: Broad-Spectrum and Selective Antifeedant

**DOI:** 10.3390/molecules31122177

**Published:** 2026-06-22

**Authors:** Liliana Ruiz-Vásquez, Maria Fe Andrés Yeves, Mao Deng Jesulin Vela Mendoza, Lastenia Ruiz Mesia, Wilfredo Ruiz Mesia, Hivelli Ricopa Cotrina, Daniel Tapia, Félix Valcarcel, Azucena Gonzalez-Coloma

**Affiliations:** 1 Laboratorio de Productos Naturales Antiparasitarios de la Amazonia, Centro de Investigación de Recursos Naturales, Universidad Nacional de la Amazonía Peruana (UNAP), Iquitos 16002, Peru; lastenia.ruiz@unapiquitos.edu.pe (L.R.M.); wilfredo.ruiz@unapiquitos.edu.pe (W.R.M.); hivelli.ricopa@unapiquitos.edu.pe (H.R.C.); 2 Facultad de Farmacia y Bioquímica, Universidad Nacional de la Amazonía Peruana (UNAP), Iquitos 16007, Peru; maovela.94@gmail.com; 3 Instituto de Ciencias Agrarias, Consejo Superior de Investigaciones Científicas (CSIC), 28006 Madrid, Spain; mafay@ica.csic.es (M.F.A.Y.); danietap@ica.csic.es (D.T.); 4 Facultad de Ingeniería Química, Universidad Nacional de la Amazonia Peruana (UNAP), Iquitos 16001, Peru; 5 Reproducción Animal, Instituto Nacional de Investigaciones Agrarias, Consejo Superior de Investigaciones Científicas, 28040 Madrid, Spain; valcarcel.felix@inia.csic.es

**Keywords:** apiol, *Piper*, essential oil, antifeedant, ixodicidal, *Hyalomma lusitanicum*, *Spodoptera littoralis*, *Myzus persicae*, methylenedioxyphenyl, integrated pest management

## Abstract

The increasing resistance of agricultural pests and disease-vectoring arthropods to synthetic pesticides underscores the urgent need for novel and sustainable biocidal agents. This study evaluates, for the first time, the insect antifeedant and ixodicidal activities of essential oils derived from ten Amazonian *Piper* species and their major constituents. Antifeedant effects were assessed against *Spodoptera littoralis*, *Myzus persicae*, *and Rhopalosiphum padi*, whereas ixodicidal activity was tested on *Hyalomma lusitanicum*. Additionally, the effects of these oils on the plant-parasitic nematode *Meloidogyne javanica* were investigated. Essential oils from *Piper mituense* (51.6% apiol) and *P. sancti-felicis* (76.1% apiol) exhibited the highest bioactivity, achieving more than 75% feeding inhibition across all insect species and 100% tick mortality. *P. mituense* consistently demonstrated greater potency, suggesting possible synergistic interactions among its minor constituents. Principal component analysis linked apiol-rich chemotypes with broad-spectrum activity. In contrast, oils rich in oxygenated caryophyllene derivatives, particularly those from *P. casapiense*, showed strong selective antifeedant effects against *R. padi*. Pure apiol displayed activity across all assays, whereas no nematicidal effects were observed. Molecular docking analyses supported these findings, indicating that apiol can interact with acetylcholinesterase in addition to its known effect on cytochrome P450 targets. Overall, these results identify complementary *Piper* chemotypes with promising potential as dual-purpose biopesticides for integrated pest management strategies.

## 1. Introduction

Resistance to synthetic pesticides in crop pests and disease-transmitting arthropods necessitates the development of alternative control strategies [1]. Key resistant species include the polyphagous lepidopteran *Spodoptera littoralis* (Boisduval), which has developed resistance to pyrethroids, organophosphates, and spinosad [2]; the aphid vector *Myzus persicae* (Sulzer), which exhibits resistance to more insecticide classes than any other herbivorous arthropod [3]; and *Rhopalosiphum padi* (L.) [4,5]. Additionally, the tick *Hyalomma lusitanicum* Koch acts as the principal reservoir and vector of Crimean–Congo hemorrhagic fever virus (CCHFV) in southwestern Europe [6,7,8,9,10]. Together, these species exemplify how multi-class resistance compromises conventional chemical control strategies [1,3,10], underscoring the urgent need to identify novel biocidal agents effective against both agricultural pests and arthropod vectors of public health concern.

Essential oils (EOs) derived from aromatic plants have emerged as promising candidates for sustainable pest management due to their rapid biodegradability, low mammalian toxicity, and multiple modes of action. These include interactions with the octopaminergic system, γ-aminobutyric acid (GABA) receptors, and acetylcholinesterase, which collectively reduce the likelihood of resistance development [11,12,13,14,15]. Among plant-based strategies, the antifeedant approach is particularly valuable within integrated pest management (IPM), as it modulates contact chemoreception rather than exerting direct lethal toxicity, thereby lowering selective pressure for resistance [16,17].

The genus *Piper* (Piperaceae), comprising approximately 2000 tropical species, produces a remarkable diversity of essential oils rich in terpenoids and phenylpropanoids with pesticidal properties [18,19,20]. Of particular interest are phenylpropanoids containing the methylenedioxyphenyl (MDP) pharmacophore, a structural motif also found in the synergist piperonyl butoxide (PBO), which inhibits cytochrome P450 monooxygenases involved in insect detoxification [21]. Dillapiole, the dominant MDP phenylpropanoid in *Piper aduncum* L., has been extensively studied for its insecticidal and acaricidal properties [22], while safrole from *P. hispidinervum* C. DC. has demonstrated antifeedant activity against *S. littoralis, M. persicae*, and *R. padi*, with documented synergistic interactions [23].

In contrast, apiol (2,5-dimethoxy-3,4-methylenedioxy-1-allylbenzene), another MDP-containing phenylpropanoid that dominates the essential oil of several *Piper* species, has received comparatively little attention despite sharing the same pharmacophore. To the best of our knowledge, neither the antifeedant nor the ixodicidal activities of apiol have been systematically evaluated, and no previous study has compared these activities across apiol-rich *Piper* chemotypes.

In a previous study, our group characterized the GC–MS composition, antifungal activity against *Botrytis cinerea*, and herbicidal potential of essential oils from ten *Piper* species collected in the Peruvian Amazon [24]. These essential oils exhibited diverse chemotypes: two species were dominated by apiol *(P. sancti-felicis*, 76.1%; *P. mituense*, 51.6%), whereas others were characterized by sesquiterpene hydrocarbons (β-bisabolene, caryophyllene, germacrene D, bicyclogermacrene) or the monoterpene limonene. Three essential oils (*P. casapiense, P. soledadense*, and *P. mituense*) were described for the first time, and dillapiole was not detected in any of the ten essential oils [24], making apiol the sole dominant MDP-bearing compound in this collection. However, the insect antifeedant and ixodicidal activities of these essential oils and their major component, apiol, were not previously investigated.

Phenylpropanoids constitute a large and structurally diverse class of plant secondary metabolites that play key roles in ecological interactions, including defense against herbivores, pathogens, and environmental stress [25,26]. Apiol, a benzodioxole-type phenylpropanoid, is widely distributed in plant essential oils and has been associated with various biological activities, including specific inhibition of aflatoxin G_1_ biosynthesis in *Aspergillus parasiticus* [27].

Accordingly, the aim of the present study was to evaluate, for the first time, the antifeedant effects of these ten Amazonian *Piper essential* oils and their major components against three model pest species (*S. littoralis, M. persicae,* and *R. padi*), as well as their ixodicidal activity against *H. lusitanicum* larvae. In addition, their effects on the plant-parasitic nematode *Meloidogyne javanica* (Treub) Chitwood were assessed. This work expands the biocidal profile of these Amazonian essential oils beyond their previously reported antifungal and herbicidal activities [24], contributing to the identification of apiol-rich *Piper* chemotypes as dual-purpose biopesticidal agents for integrated pest and tick management. Furthermore, molecular docking, an established computational method that predicts ligand–protein interactions by sampling conformations and ranking them using scoring functions [28,29], was employed to compare the binding interactions of apiol, a known P-450 enzymatic inhibitor, with those of known ligands targeting acetylcholinesterase and cytochrome P450 proteins in insects, ticks, and nematodes [30]. This approach provides further insight into the potential mechanisms of action of apiol and apiol-rich essential oils.

## 2. Results

### 2.1. Essential Oil Composition

Table 1 shows the summarized chemical composition of the *Piper* essential oils studied here, determined by GC-MS. Their chemical composition was previously reported [24]. Based on their dominant chemical classes, the essential oils were classified into four chemotypes: (i) a phenylpropanoid chemotype dominated by apiol (*P. sanctifelicis*, 76.1%; *P. mituense*, 51.6%); (ii) a predominantly monoterpene chemotype rich in limonene (*P. soledadense*, 38.5%); (iii) a bisabolene-type sesquiterpene chemotype (*P. coruscans*, β-bisabolene 33.4%; *P. tuberculatum*, β-bisabolene 40.2%); and (iv) a mixed sesquiterpene chemotype characterized by varying proportions of caryophyllenes (*P. anonifolium*), germacrene D, and bicyclogermacrene (*P. obliquum*, *P. dumosum*, *P. reticulatum*, and *P. casapiense*). In this work, additional tentative chemical identification of previously unidentified compounds has been carried out based on retention index and EI mass spectrum (see Table S1 in Supplementary Information).

Three major (>5%) compounds have been identified in *P. casapiense*. Compounds eluting at rt 19.49 and 21.13 min (RI = 1711 and 1798) were dominated by ions at *m*/*z* 93, 91, 105 and 133, characteristic of caryophyllane-type fragmentation. These compounds have been tentatively identified as caryophyllenone and β-caryophyllenol. A third oxygenated caryophyllane derivative (rt 21.55 min) was detected with a retention index of 1821. Its EI mass spectrum was dominated by characteristic caryophyllane ions at *m*/*z* 93, 91, 105 and 133, together with additional fragments at *m*/*z* 107, 119 and 121, indicative of a higher degree of oxygenation. Based on its elevated retention index and fragmentation pattern, this compound was tentatively identified as a caryophyllane-type diol or highly oxygenated caryophyllenol derivative. Its co-occurrence with caryophyllenone and β-caryophyllenol supports a sequential oxidative transformation of a common β-caryophyllene precursor within the essential oil. Similarly, in *P. anonifolium*, the EI mass spectrum (*m*/*z* 161/105/81/204/119/162/134/91/159/131) of a compound eluting at rt 15.32 min and RI 1503 was characteristic of a caryophyllane alcohol. Additionally, in *P. obliquum*, the EI mass spectrum (*m*/*z* 95/121/161/204/43/109/105/81/164/108) of the compound eluting at rt 18.46 min and RI 1658 was characteristic of a highly oxygenated caryophyllane-type sesquiterpene (diol derivative).

### 2.2. Bioactivity

The insect antifeedant effects of the tested essential oils are shown in Table 2. The most sensitive insects were the aphids *M. persicae* (7 active of 10), *R. padi* (6 active of 10) and the lepidopteran *S. littoralis* (3 active of 10). Regarding insect species selectivity, *P. mituense* and *P. sancti-felicis* were active against all three insect species; *P. anonifolium, P. casapiense*, *P. obliquum* and *P. reticulatum* had significant effects on both aphid species; *P. coruscans* and *P. tuberculatum* were only active on *M. persicae;* and *P. soledadense* was only active on *R. padi*. The most active oil on *R. padi* was *P. casapiense* (EC_50_, 3.8 µg/cm^2^), followed by *P. sancti-felicis* (EC_50_, 8.6 µg/cm^2^), *P. mituense* (EC_50_, 9.7 µg/cm^2^) and *P. obliquum* (EC_50_, 25.6 µg/cm^2^), while *P. soledadense* and *P. anonifolium* had a significant effect but did not reach the 70% inhibition threshold to carry out dose–response experiments. On *M. persicae, P. reticulatum* was the most active (EC_50_, 16.2 µg/cm^2^) followed by *P. mituense* (EC_50_, 23.2 µg/cm^2^), *P. tuberculatum* (EC_50_, 39.5 µg/cm^2^) and *P. sancti-felicis* (EC_50_, 53.5 µg/cm^2^), while *P. anonifolium, P. casapiense, P. coruscans* and *P. obliquum* had a significant effect but did not reach the 70% inhibition threshold to carry out dose–response experiments. On *S. littoralis*, *P. mituense* (EC_50_, 15.6 µg/cm^2^) was very active, followed by *P. sancti-felicis* (EC_50_, 42.0 µg/cm^2^) and *P. dumosum*, with a feeding inhibition value below the 70% inhibition threshold.

Among the major components of the oils (Table 1), the following compounds were tested: apiol, β-bisabolene, δ-cadinene, β-caryophyllene, β-caryophyllene oxide, germacrene D, α-humulene, limonene, nerolidol, β-pinene, and α-pinene (Table 2). Additionally, myristicin was included because of its potential synergistic action.

Apiol was active against the three insect targets, with EC_50_ values of 22.24, 16.00 and 17.70 µg/cm^2^ against *S. littoralis, M. persicae* and *R. padi* respectively. β-Bisabolene and myristicin were active on *R. padi*, with EC_50_ values of 26.51 and 25.50 µg/cm^2^ respectively. Nerolidol showed significant effects on both aphid species and α-pinene on *S. littoralis* but did not reach the 70% inhibition threshold to carry out dose–response experiments. Thymol was included as a positive control with EC_50_ values of 21.00, 7.60 and 18.60 µg/cm^2^ against *S. littoralis, M. persicae* and *R. padi* respectively (Table 2).

The ten essential oils and apiol were also evaluated for nematicidal activity against *Meloidogyne javanica* second-stage juveniles (J2). None of the essential oils or the pure compounds exhibited significant nematicidal effects at the concentrations tested, except for the positive control thymol (see Table S2).

Table 3 shows the ixodicidal activity of the ten *Piper* essential oils against *Hyalomma lusitanicum* larvae. Five essential oils were effective ixodicidal agents, with *P. mituense* being the most active and similar to the positive control thymol (LD_50_ = 1.46 µg/mg cellulose, thymol = LD_50_ of 1.47 µg/mg cellulose), followed by *P. sanctifelicis* (LD_50_ = 2.14 µg/mg cellulose), *P. reticulatum* (LD_50_ = 6.19 µg/mg cellulose), *P. dumosum* (LD_50_ = 8.89 µg/mg cellulose), and *P. soledadense* (LD_50_ = 10.05 µg/mg cellulose).

The major components of the active oils (apiol, limonene, α- and β-pinene, β-caryophyllene, β-caryophyllene oxide, nerolidol, copaene and δ-cadinene) and myristicin were tested on *H. lusitanicum* larvae. Thymol was included as a positive control (Table 3). Among the compounds tested, apiol (LD_50_ of 1.96 µg/mg cellulose), β-caryophyllene oxide (LD_50_ of 4.46 µg/mg cellulose) and myristicin (LD_50_ of 2.88 µg/mg cellulose) were active, with lower potency levels than the positive control thymol (Table 3).

#### 2.2.1. Principal Component Analysis

**Figure 1:**
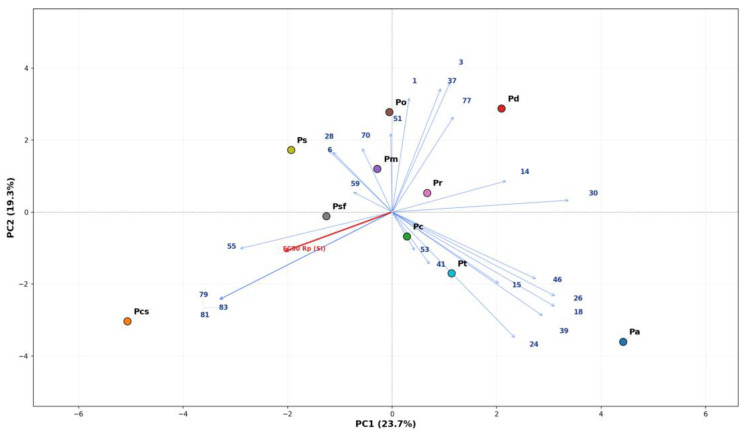
PCA biplot integrating the chemical composition and antifeedant activity against *Rhopalosiphum padi* (Sl) of essential oils from ten Amazonian *Piper* species (23 compounds with relative abundance ≥5% and EC_50_; autoscaled). Blue arrows represent compound loadings; red arrows represent EC_50_ values, with direction inverted so that the vectors point toward the most active species (lower EC_50_ = higher activity). PC1 and PC2 explain 23.7% and 19.3% of the total variance, respectively (43.0% cumulative). Species abbreviations: Pa = *P. anonifolium*; Pcs = *P. casapiense*; Pc = *P. coruscans*; Pd = *P. dumosum*; Pm = *P. mituense*; Po = *P. obliquum*; Pr = *P. reticulatum*; Psf = *P. sancti-felicis*; Ps = *P. soledadense*; Pt = *P. tuberculatum*. Compound numbers correspond to their elution order in the GC-MS database (Table S1): **1** = α-pinene; **3** = β-pinene; **6** = limonene; **14** = α-cubebene; **15** = cis-β-copaene; **18** = β-caryophyllene; **24** = α-humulene; **26** = neoalloocimene; **28** = γ-muurolene; **30** = germacrene D; **37** = bicyclogermacrene; **39** = caryophyllane alcohol isomer II; **41** = β-bisabolene; **46** = δ-cadinene; **51** = 10-epi-elemol; **53** = nerolidol; **55** = caryophyllene oxide; **59** = apiol; **70** = highly oxygenated caryophyllane-type sesquiterpene diol derivative; **77** = dillapiol; **79** = caryophyllenone; **81** = β-caryophyllenol; **83** = caryophyllane-type diol.

Principal component analysis (PCA) was performed on a matrix comprising 26 active variables (23 compounds with relative abundance ≥ 5% in at least one species and three antifeedant EC_50_ values) across ten *Piper* species, using autoscaling. The first two principal components accounted for 21.9% and 18.3% of the total variance, respectively (40.2% cumulative), and their biplot is shown in Figure 1.

The score plot revealed three distinct groupings. The apiol-rich species *P. mituense* (Pm) and *P. sancti-felicis* (Psf) clustered in the upper-left quadrant, driven by the strong positive loading of apiol on PC2 and its negative contribution to PC1. In contrast, the β-bisabolene-dominated species *P. coruscans* (Pc) and *P. tuberculatum* (Pt) were projected into the lower-central region, associated with the loadings of β-bisabolene, δ-cadinene, nerolidol, and β-caryophyllene. The sesquiterpene-rich species *P. anonifolium* (Pa) and *P. dumosum* (Pd) occupied the right side of the plot, separated along PC1 by high loadings of germacrene D, α-cubebene, and α-humulene.

*P. casapiense* (Pcs) was clearly isolated in the lower-left quadrant, driven by oxygenated caryophyllane derivatives (caryophyllenone, β-caryophyllenol, caryophyllane oxide, and oxygenated caryophyllane diol), which are exclusive to this species. *P. soledadense* (Ps) occupied an intermediate position in the left-central region, consistent with its limonene-dominated monoterpene chemotype. *P. obliquum* (Po) and *P. reticulatum* (Pr) were positioned near the center, reflecting their mixed sesquiterpene profiles without a single dominant compound.

The projection of the antifeedant EC_50_ variables (red arrows, Figure 1) provided insight into the relationship between chemical composition and biological activity. The EC_50_ vectors for *S. littoralis* (Sl) and *M. persicae* (Mp) pointed toward the center-right of the plot, while the EC_50_ vector for *R. padi* (Rp) was directed toward the lower-left quadrant. Since lower EC_50_ values indicate greater antifeedant potency, the most active species are those projected in the direction opposite to the EC_50_ vectors. Accordingly, Pm and Psf, located opposite all three EC_50_ vectors, were associated with the strongest broad-spectrum antifeedant activity, consistent with their apiol-rich chemotype. The proximity of the apiol loading vector to these species, together with its opposition to the EC_50_ vectors, confirms that apiol content is the primary chemical determinant of broad-spectrum antifeedant efficacy.

Conversely, the sesquiterpene-dominated species (Pa, Pc, and Pt) were projected near or along the EC_50_ vectors, consistent with their weak or absent antifeedant effects. *P. casapiense* (Pcs), despite its position opposite the Rp vector, showed strong selective activity against *R. padi* (EC_50_ = 3.8 µg/cm^2^), suggesting that its oxygenated caryophyllane derivatives may contribute to taxon-selective antifeedant effects.

**Figure 2:**
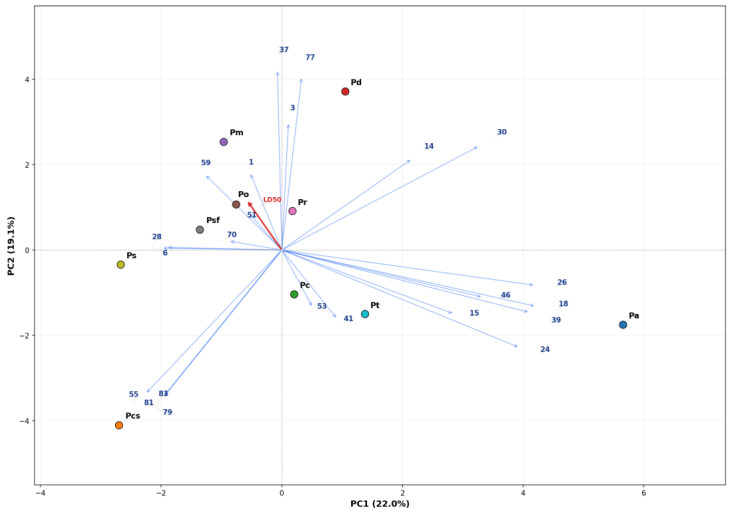
PCA biplot integrating the chemical composition and ixodicidal activity (LD_50_) against *Hyalomma lusitanicum* larvae of essential oils from ten Amazonian *Piper* species (24 active variables: 23 compounds with relative abundance ≥5% + LD_50_; autoscaled). Blue arrows represent compound loadings; the red arrow represents the LD_50_, with direction inverted so that the vector points toward the most active species (lower LD_50_ = higher ixodicidal activity). PC1 and PC2 explain 22.0% and 19.1% of the total variance, respectively (41.1% cumulative). Species abbreviations: Pa = *Piper anonifolium*; Pcs = *P. casapiense*; Pc = *P. coruscans*; Pd = *P. dumosum*; Pm = *P. mituense*; Po = *P. obliquum*; Pr = *P. reticulatum*; Psf = *P. sancti-felicis*; Ps = *P. soledadense*; Pt = *P. tuberculatum*. Compound numbers correspond to their elution order in the GC-MS analysis (Table S1): **1** = α-pinene; **3** = β-pinene; **6** = limonene; **14** = α-cubebene; **15** = cis-β-copaene; **18** = β-caryophyllene; **24** = α-humulene; **26** = neoalloocimene; **28** = γ-muurolene; **30** = germacrene D; **37** = bicyclogermacrene; **39** = caryophyllane alcohol isomer II; **41** = β-bisabolene; **46** = δ-cadinene; **51** = 10-epi-elemol; **53** = nerolidol; **55** = caryophyllene oxide; **59** = apiol; **70** = highly oxygenated caryophyllane-type sesquiterpene diol derivative; **77** = dillapiol; **79** = caryophyllenone; **81** = β-caryophyllenol; **83** = caryophyllane-type diol.

A second PCA (Figure 2) was performed on a matrix of 24 active variables (23 compounds with relative abundance ≥5% in at least one species and the LD_50_ against *H. lusitanicum* larvae) across the ten *Piper* species, using autoscaling. PC1 and PC2 explained 22.0% and 19.1% of the total variance, respectively (41.1% cumulative).

The LD_50_ vector (red arrow) pointed toward the upper-left quadrant, where the five ixodicidally active species were clustered: *P. mituense* (Pm, LD_50_ = 1.46 µg/mg), *P. sancti-felicis* (Psf, LD_50_ = 2.14 µg/mg), *P. reticulatum* (Pr, LD_50_ = 6.19 µg/mg), *P. dumosum* (Pd, LD_50_ = 8.89 µg/mg), and *P. soledadense* (Ps, LD_50_ = 10.06 µg/mg). The apiol loading vector was co-directional with the LD_50_ vector and closely aligned with Pm and Psf, the two most potent species, confirming that apiol is the primary driver of ixodicidal activity. This interpretation is consistent with the high potency of pure apiol (LD_50_ = 1.97 µg/mg, Table 3).

Notably, Pd was projected in the upper quadrant alongside the dillapiol, bicyclogermacrene, and β-pinene vectors, suggesting that its ixodicidal activity (100% mortality) may be attributable to dillapiol (8.9%) and myristicin (3.7%), both methylenedioxyphenyl (MDP) compounds, rather than to its sesquiterpene background. This contrasts with the antifeedant PCA (Figure 1), in which Pd failed to exhibit broad-spectrum activity, indicating that the threshold of MDP-phenylpropanoids required for ixodicidal effects may be lower than that needed for antifeedant efficacy.

In contrast, the inactive species (LD_50_ > 40 µg/mg) were projected in the opposite region of the biplot. *P. anonifolium* (Pa) was isolated on the far right, driven by the loadings of α-humulene, β-caryophyllene, caryophyllane alcohol isomer II, and neoalloocimene, whereas *P. coruscans* (Pc) and *P. tuberculatum* (Pt) clustered in the lower-central region along the β-bisabolene and δ-cadinene vectors. Their position opposite the LD_50_ vector is consistent with the lack of ixodicidal activity observed for pure β-caryophyllene, limonene, α-pinene, β-pinene, and copaene (all with LD_50_ > 20 µg/mg, Table 3).

*P. casapiense* (Pcs) was again isolated in the lower-left quadrant due to its exclusive oxygenated caryophyllane derivatives (caryophyllenone, β-caryophyllenol, and caryophyllene oxide). Despite showing moderate mortality (53 ± 7%), it did not yield a calculable LD_50_, indicating that the oxygenated caryophyllane chemotype, responsible for potent, selective antifeedant activity against *R. padi* (Section 2.2.1), does not translate into equivalent ixodicidal effects. Among the non-apiol compounds, only β-caryophyllene oxide (LD_50_ = 4.46 µg/mg) and myristicin (LD_50_ = 2.89 µg/mg) exhibited notable activity. Their individual contributions may partially explain the ixodicidal effects observed in Ps (caryophyllene oxide, 13.3%) and Pr (apiol, 15.0%, combined with a mixed sesquiterpene profile).

#### 2.2.2. Docking

**Figure 3:**
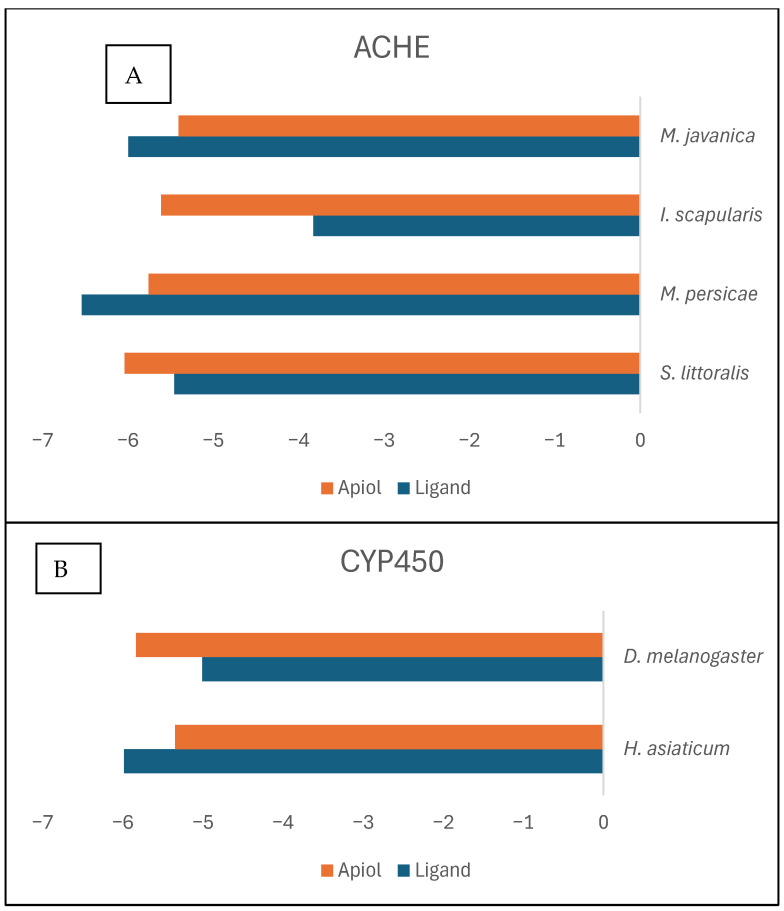
(**A**) Comparative docking scores of apiol and acetylcholine in acetylcholinesterase (AChE) from four target species. Bar plots represent the best (lowest) docking scores obtained from 50 generated poses for each ligand–protein pair. Species included are *Spodoptera littoralis, Myzus persicae, Ixodes scapularis*, and *Meloidogyne javanica*. More negative values indicate stronger predicted binding affinity. (**B**) Docking score comparison of apiol and reference ligands in cytochrome P450 enzymes. Bar plots show the best docking scores for apiol and species-specific reference ligands: terpinolene for *Hyalomma asiaticum* CYP3A8 and α-pinene for *Drosophila melanogaster* CYP6A2. Values correspond to the most favorable pose among 50 generated conformations.

Apiol demonstrated binding affinities comparable to or better than those of reference ligands across several targets (Figure 3, Supplementary Table S3). In AChE enzymes, improved binding was observed in *S. littoralis* and *Ixodes scapularis*, whereas slightly reduced affinity was found in *M. persicae* and *M. javanica*. In CYP450 enzymes, apiol showed improved binding in *Drosophila melanogaster* relative to α-pinene but slightly reduced affinity in *H. asiaticum* compared to terpinolene.

Apiol maintained interactions with catalytic residues in AChE. The interactions with the Ser and His of the catalytic triad were consistent across the generated poses. Also, the same interactions of acetylcholine with non-catalytic residues were detected in the case of apiol, indicating proper positioning within the active site (Supplementary Table S1). Additional interactions not observed in control ligands were identified. In the case of *D. melanogaster*’s CYP450, only the interaction with the catalytic Ile was detected; nonetheless, other non-catalytic interactions were shared between the reference ligand and apiol, suggesting the compatibility of the molecule with the binding position.

## 3. Discussion

The present study demonstrates that apiol-rich essential oils from Amazonian *Piper* species exhibit strong and consistent biocidal activity across both insect pests and ticks, supporting their potential as dual-purpose agents for integrated pest management. The broad-spectrum antifeedant activity observed for *P. mituense* and *P. sancti-felicis*, together with their high ixodicidal efficacy and the strong correlation between apiol content and biological activity revealed by PCA, highlights the central role of apiol as a key determinant of bioactivity. This finding is consistent with the ecological function of phenylpropanoids in plant defense, where these metabolites contribute to herbivore deterrence and protection against biotic stress. Notably, many phenylpropanoids act as synergists by interfering with detoxification pathways, including the inhibition of cytochrome P450-mediated enzymatic systems [25,26].

A notable exception to the apiol-driven pattern was *P. casapiense*, which exhibited the strongest selective antifeedant activity against *R. padi* despite lacking high apiol content. This species, characterized by a chemotype rich in oxygenated caryophyllene derivatives (β-caryophyllenol, caryophyllenone, and other highly oxygenated caryophyllane-type compounds), was clearly separated in the PCA and positioned opposite to the *R. padi* EC_50_ vector. This suggests that its bioactivity is governed by a chemical mechanism distinct from that of apiol-rich essential oils. Oxygenated caryophyllane sesquiterpenes, especially caryophyllene oxide, are widely reported to exhibit multi-target bioactivity against insects, including larvicidal effects, oviposition deterrence, developmental disruption, and strong repellency, often exceeding the activity of the non-oxygenated parent compound, β-caryophyllene [32,33,34,35]. A similar pattern was observed for *P. obliquum*, characterized by the presence of bicyclogermacrene, oxygenated caryophyllene derivatives, and related sesquiterpenes. Bicyclogermacrene has been reported to show potent larvicidal activity against mosquito species such as *Anopheles subpictus*, *Aedes albopictus*, and *Culex tritaeniorhynchus* [36]. Sesquiterpene-rich essential oils are also known to interfere with insect feeding behavior and sensory perception, particularly in aphids, which rely heavily on contact chemoreception [16,17]. Therefore, in addition to apiol-mediated broad-spectrum activity, alternative chemotypes based on oxygenated caryophyllenes and related sesquiterpenes may confer potent but more selective antifeedant effects.

The observed differences between antifeedant and ixodicidal responses further suggest the involvement of distinct biological targets and activity thresholds. While high apiol content appears necessary to achieve broad-spectrum antifeedant activity, ixodicidal effects were observed at lower concentrations, as illustrated by the activity of *P. dumosum*, which contains moderate levels of methylenedioxyphenyl (MDP)-bearing compounds such as dillapiol and myristicin. At the molecular level, docking results provide mechanistic support for these experimental observations. Apiol exhibited binding affinities comparable to, or greater than, those of endogenous ligands for acetylcholinesterase (AChE), suggesting a potential role on this target. AChE plays a central role in neurotransmission by hydrolyzing acetylcholine, and its inhibition leads to the accumulation of the neurotransmitter and disruption of neural signaling, ultimately resulting in paralysis and death in arthropods [37,38]. 

Apiol also showed relevant interactions with cytochrome P450 enzymes. Inhibition of cytochrome P450 monooxygenases is a mechanism well documented for methylenedioxyphenyl (MDP) phenylpropanoids such as apiol [39]. The variability observed across CYP450 targets reflects the structural diversity and functional adaptability of these enzymes, which are known to metabolize a wide range of xenobiotics [40]. Enhanced CYP450 activity is a major mechanism of insecticide resistance, allowing insects to degrade toxic compounds efficiently [41,42], similarly to piperonyl butoxide, a well-known synergist that inhibits cytochrome P450-mediated detoxification pathways in insects [21,43]. 

The integration of chemical composition, bioactivity, and molecular docking data allows for the identification of clear structure–activity relationships (SAR) within the essential oils of *Piper* species. Two major chemotypes emerge as biologically relevant: (i) apiol-rich phenylpropanoid systems, associated with broad-spectrum activity, and (ii) sesquiterpene-rich systems, particularly those dominated by oxygenated caryophyllenes, associated with selective antifeedant effects. Taken together, these findings support a dual SAR framework in *Piper* essential oils: (i) MDP-phenylpropanoids (apiol, dillapiol-like systems) confer broad-spectrum activity through a possible action on AChE while modulating CYP450 involved in detoxification; (ii) oxygenated sesquiterpenes (caryophyllene derivatives) confer selective antifeedant activity through behavioral and sensory interference mechanisms. This duality provides a mechanistic explanation for the diversity of biological responses observed and highlights the potential of combining different chemotypes to achieve both broad-spectrum and target-specific pest control strategies.

## 4. Materials and Methods

### 4.1. Plant Material and Essential Oil Extraction

The aerial parts (leaves, stems, and flowers) of ten *Piper* species were collected in three districts of the Loreto Department, Peruvian Amazon: Mazán District (*P. coruscans*, voucher 039849; *P. casapiense*, 041044; *P. obliquum*, 027690; *P. anonifolium*, 042381; *P. tuberculatum*, 020115), Punchana District (*P. sancti-felicis*, 036367), and San Juan Bautista District (*P. dumosum*, 040311; *P. reticulatum*, 042127; *P. soledadense*, 033308; *P. mituense*, 041473). Voucher specimens were deposited at the Herbarium AMAZ, Universidad Nacional de la Amazonia Peruana (UNAP), Iquitos, Peru. All plant material was collected under Regional Management Resolution No. 035, 2021, GRL, GGR, GRDFFS. Essential oils were obtained by hydrodistillation of the dried aerial parts, separated by decantation, and dried over anhydrous Na_2_SO_4_ (Sigma-Aldrich, St. Louis, MO, USA), yielding between 0.078% and 1.26% (dry weight basis). Collection coordinates, dry weights, and detailed extraction procedures have been previously reported [24].

### 4.2. Gas Chromatography–Mass Spectrometry (GC-MS) Analysis

The chemical composition of the essential oils was determined by GC–MS using a Shimadzu GCMS-QP2010 Ultra (Shimadzu, Kyoto, Japan), Ultra system equipped with a TRB-5 capillary column (Teknokroma, Barcelona, Spain; 30 m × 0.25 mm ID, 0.25 µm film thickness) under the conditions previously described [24]. Compound identification was based on comparison of mass spectra with the Wiley 275 Mass Spectral Database and retention indices from the literature. Quantification was performed using relative peak area percentages. The complete chemical profiles of the ten essential oils, including three species described for the first time (*P. soledadense*, *P. casapiense*, and *P. mituense*), are detailed in [24] and summarized in Table 1 of the present work.

### 4.3. Antifeedant Bioassays

Insect colonies of *Spodoptera littoralis* (Boisduval), *Myzus persicae* (Sulzer), and *Rhopalosiphum padi* (L.) were maintained at the Instituto de Ciencias Agrarias (ICA-CSIC, Madrid, Spain). *S. littoralis* was reared on artificial diet, *M. persicae* on bell pepper (*Capsicum annuum* L.), and *R. padi* on barley (*Hordeum vulgare* L.). All colonies were kept in a custom-made walk-in growth chamber at 22 ± 1 °C, >70% relative humidity (RH), and a 16:8 h light:dark (L:D) photoperiod [44].

Antifeedant activity was evaluated by dual-choice feeding/settling tests [44]. The upper surface of leaf disks or leaf fragments (1.0 cm^2^) of the corresponding host plant was treated with the test solution (essential oils). For each comparison, paired disks/fragments were offered simultaneously: one treated with the test substance dissolved in solvent (treatment) and the other treated with solvent alone (control).

Lepidopteran assay (*S. littoralis*). Tests were carried out with newly molted sixth-instar (L6) larvae (>24 h after molting). Each *C. annuum* leaf disk (1.0 cm^2^) was surface-treated with 10 µL of the test solution. Six Petri dishes (replicates) were used per treatment, each containing two treated disks, two control disks, and two L6 larvae. Larvae were allowed to feed under the rearing conditions described above until ≈75% of the control disk surface was consumed.

Aphid assays (*M. persicae* and *R. padi*). Apterous adults (24–48 h old) were used. Ventilated plastic boxes (3 × 3 × 1.5 cm) were each lined with a thin layer of 2.5% agar to maintain leaf turgor and prevent desiccation. For *M. persicae*, two half-disks of *C. annuum* leaves were used per box; for *R. padi*, two barley (*H. vulgare*) leaf fragments (1.0 cm^2^ each) were used. One half/fragment was treated with the test substance and the other with solvent (control), each receiving 10 µL of the corresponding solution. Twenty boxes (replicates) were used per treatment, with 10 aphids per box. Boxes were incubated inverted under indirect light at 22 ± 1 °C and a 16:8 h L:D photoperiod for 24 h, after which the number of aphids settled on the treated and control surfaces was recorded.

Essential oils were assayed at an initial (baseline) dose of 100 µg/cm^2^ (10 µg/µL × 10 µL). Pure compounds, including apiol (Sigma-Aldrich, St. Louis, MO, USA) and the additional major constituents tested, were assayed at an initial dose of 50 µg/cm^2^ (5 µg/µL × 10 µL) under identical conditions. Each full experiment was repeated twice [44].

For *S. littoralis*, the percent feeding inhibition was calculated as %FI = [1 − (T/C)] × 100, where T and C are the leaf-disk areas consumed in the treatment and control, respectively; consumed areas were measured by image analysis (ImageJ v1.54, National Institutes of Health, Bethesda, MD, USA; https://imagej.nih.gov/ij/, accessed on 15 January 2026). For the aphids, the percent settling inhibition was calculated as %SI = [1 − (%T/%C)] × 100, where %T and %C are the percentages of aphids settled on the treated and control surfaces, respectively. Significance testing, the activity threshold used to trigger dose–response experiments, and the estimation of effective doses (EC_50_) are described in Section 4.6. 

### 4.4. Nematicidal Bioassays

A population of the root-knot nematode *Meloidogyne javanica* (Treub) Chitwood was maintained at ICA-CSIC (Madrid, Spain) on tomato (*Solanum lycopersicum* L., var. Marmande) plants in pot cultures at 25 °C and 70% RH [45]. Egg masses were hand-picked from infected tomato roots, and second-stage juveniles (J2) were obtained by incubating the hand-picked egg masses as a water suspension at 25 °C for 24 h.

The in vitro effect on juveniles was evaluated in 96-well plates. Test solutions were prepared by dissolving each essential oil or pure apiol in a DMSO–Tween solution (0.5% Tween 20 in DMSO; Sigma-Aldrich, St. Louis, MO, USA). For each well, 5 µL of test solution was added to 95 µL of a water suspension containing approximately 100 J2, giving an initial (baseline) test concentration of 1 µg/µL (1 mg/mL) [45]. Four control wells containing the water/DMSO–Tween solution without test substance were included in each experiment. All treatments were assayed in quadruplicate. Plates were covered to prevent evaporation and incubated in darkness at 25 °C. Dead J2 were counted under a Nikon SMZ745 binocular microscope (Nikon Corporation, Tokyo, Japan) after 72 h, scoring juveniles as dead when they remained immobile and straight after mechanical stimulation. Percent mortality was corrected against the negative control using Schneider–Orelli’s formula [13].

### 4.5. Ixodicidal Bioassays

Engorged *Hyalomma lusitanicum* Koch females were collected from red deer (*Cervus elaphus*) in Ciudad Real (Central Spain) and maintained under laboratory conditions (22–24 °C, 80% RH) until oviposition and egg hatching. Larvae older than 6 weeks were used for the bioassays [44,46]. 

Larvicidal activity was determined by the impregnated-substrate (cellulose) method [44,46]. For each concentration, 50 µL of the test solution was applied to 25 mg of powdered crystalline cellulose (Sigma-Aldrich, St. Louis, MO, USA), and the solvent (acetone, Sigma-Aldrich, St. Louis, MO, USA) was allowed to evaporate completely. The essential oils were assayed at an initial (baseline) dose of 20 µg/mg cellulose and the pure compounds at 10 µg/mg cellulose. Batches of 20 larvae were then transferred onto the treated cellulose; the tubes were plugged with hydrophilic cotton and gently rotated several times to homogenize the larvae–cellulose mixture and ensure full contact. Three replicates (20 larvae each) were used per treatment and concentration. Tubes were kept under laboratory conditions (22–24 °C, >70% RH) for 24 h.

Negative controls (cellulose treated with solvent only) and positive controls were processed in parallel and in triplicate. Thymol (20 µg/mg cellulose; Sigma-Aldrich, St. Louis, MO, USA) was used as the reference positive control [44].

Mortality was assessed after 24 h of contact with the treated cellulose using a binocular magnifying glass; larvae were scored as dead when unable to move from one place to another. Percent mortality was corrected relative to the negative control using Schneider–Orelli’s formula [13]: %M = [(%T − %C)/(100 − %C)] × 100, where %T and %C are the percentages of dead larvae in the treatment and negative control, respectively.

### 4.6. Statistical Analysis

Antifeedant effects (%FI and %SI) were analyzed for significance using the non-parametric Wilcoxon signed-rank test. Effective antifeedant concentrations (EC_50_) were determined by linear regression analysis (%FI or %SI vs. log dose) for essential oils exhibiting inhibition values ≥ 75% (Table 2). The larvicidal activity data are presented as percent mortality corrected according to Schneider–Orelli’s formula [13]. Effective lethal doses (LD_50_ and LD_90_) were calculated by Probit analysis (1:2 serial dilutions covering a range of activity between 100% and <50% mortality, with a minimum of three doses) (Table 3). Both EC_50_ and Probit analyses were performed using STATGRAPHICS Centurion XVI (version 16.1.02).

Principal component analyses (PCA) were performed to explore the relationships between essential oil composition and biological activity. The antifeedant PCA was performed on a matrix of 26 active variables: the 23 compounds with relative abundance ≥5% in at least one species and the three antifeedant EC_50_ values (against *S. littoralis, M. persicae*, and *R. padi*). The ixodicidal PCA was performed on a matrix of 24 active variables: the same 23 compounds and the LD_50_ against *H. lusitanicum* larvae. All variables were autoscaled prior to analysis. PCA analyses and visualizations were performed in Python v3.11.6 (Python Software Foundation, Wilmington, DE, USA) on the Julius AI platform (Julius 1.2 Max; Julius AI, Inc., San Francisco, CA, USA; accessed on 6 March 2026) on the Julius computational platform (https://julius.ai/). 

### 4.7. Molecular Docking

The protein sequences of acetylcholinesterase were obtained from the UniProt database. The entries chosen were B7QEQ1 (*Ixodes scapularis*), Q8T7U9 (*Myzus persicae*), A0A915MGH7 (*Meloydogine javanica*) and A0A9P0N5I4 (*Spodoptera littoralis*) (https://doi.org/10.1093/nar/gkae1010). The protein CYP450 3A8 of *Hyalomma asiaticum* was obtained from Genbank (https://doi.org/10.1093/nar/gks1195) (entry: PQ058615.1), and the protein CYP450 6A2 of *Drosophila melanogaster* was obtained from UniProt (entry: P33270). All the 3D models were generated using the SWISS-MODEL tool (https://doi.org/10.1093/nar/gky427).

Ligands were prepared using the OPLS4 force field, which provides enhanced accuracy in modeling molecular interactions and conformational energetics [30]. Ionization states were assigned using Epik, and stereochemistry was preserved. Protein structures were prepared at physiological pH (7.4). Hydrogen atoms were added and protonation states optimized to ensure proper representation of electrostatic interactions.

Docking grids were centered on active-site residues with a grid size of 20 Å. Fifty poses were generated for each ligand–protein pair and ranked based on docking score. Docking predicts the preferred binding orientation and affinity of ligands to protein targets through scoring functions [28]. The best (lowest) docking score per system was selected. Interaction patterns were analyzed across all generated poses to identify conserved residues and unique contacts. Interaction types were assigned based on residue chemical properties and ligand orientation, considering standard criteria (hydrogen bonding, π–π stacking, hydrophobic and polar interactions) derived from docking poses.

### 4.8. Use of Generative Artificial Intelligence

In preparing this manuscript, the authors utilized AI-assisted tools (Claude Opus 4.6, Anthropic, San Francisco, CA, USA) to enhance the grammatical structure and linguistic clarity of the English writing, and PCA visualizations were generated in Python v3.11.6 with the assistance of Julius AI (San Francisco, CA, USA). All scientific content, experimental design, data collection, analysis, and interpretation represent the original intellectual contribution of the research team. No AI-assisted tools were used for data generation, scientific reasoning, or interpretation of results. The authors reviewed and take full responsibility for the accuracy and integrity of all content presented in this publication.

## 5. Conclusions

This study demonstrates, for the first time, that apiol-rich essential oils from Amazonian *Piper* species exhibit dual biopesticidal activity: broad-spectrum antifeedant effects against *S. littoralis*, *M. persicae*, and *R. padi* (EC_50_ 0.38–5.35 µg/cm^2^), together with potent ixodicidal activity against *H. lusitanicum* (LD_50_ 0.046–0.267 µg/mg cellulose). Among the ten species evaluated, *P. mituense* and *P. sancti-felicis*, both dominated by the phenylpropanoid apiol, were the only essential oils that exceeded the 70% inhibition threshold against all three pest species while simultaneously achieving 100% tick larval mortality under the tested conditions.

The consistently higher potency of *P. mituense* compared with *P. sancti-felicis* and pure apiol across all bioassays suggests synergistic contributions from minor constituents, particularly myristicin, bicyclogermacrene, and germacrene D. Principal component analysis further indicates that the chemical class of the dominant constituent influences biocidal selectivity: apiol-rich chemotypes displayed the strongest broad-spectrum activity, whereas sesquiterpene-dominated oils produced more taxon-selective effects of lower overall magnitude. Furthermore, molecular docking results indicated that apiol can establish favorable interactions with acetylcholinesterase in addition to cytochrome P450 enzymes in arthropods.

Overall, these results address an important knowledge gap, as apiol has been scarcely investigated as an antifeedant or ixodicidal agent. They also expand the known biocidal profile of Amazonian *Piper* chemotypes beyond previously reported antifungal and herbicidal activities, highlighting apiol-rich essential oils as promising dual-purpose candidates for the integrated management of crop pests and arthropod vectors of public health importance.

## Figures and Tables

**Table 1:** Major components (≥5%) of the essential oils from ten Amazonian *Piper* species.

Species	Major Components (≥5%)
*P. anonifolium*	β-Caryophyllene (11.3%), germacrene D (9.6%), caryophyllane-type sesquiterpene (9.2%), δ-cadinene (6.6%), α-humulene (6.6%), (−)-β-copaene (5.8%), *cis*-β-copaene (5.8%), neoalloocimene (5.5%)
*P. casapiense*	β-Caryophyllenol (22%), β-caryophyllene oxide (10.2%), caryophyllane-type diol (5.4%)
*P. coruscans*	β-Bisabolene (33.4%), nerolidol (10.2%), β-caryophyllene (8%), (+)-β-Selinene (5%)
*P. dumosum*	Bicyclogermacrene (16.5%), germacrene D (10.4%), dillapiol (8.9%), β-caryophyllene (6.8%), β-pinene (6.3%), α-cubebene (5.9%), myristicin (3.7%)
*P. mituense*	Apiol (51.6%), bicyclogermacrene (9.0%), germacrene D (6.7%), dillapiol (5.3%), myristicin (4.6%)
*P. obliquum*	Bicyclogermacrene (7.9%), highly oxygenated caryophyllane-type sesquiterpene (7.7%), 10-epi-elemol (7.3%), caryophyllene oxide (6.3%), β-caryophyllene (6.3%), α-pinene (6%), β-pinene (5.1%)
*P. reticulatum*	Apiol (15%), germacrene D (12.6%), bicyclogermacrene (8.1%), δ-cadinene (6%)
*P. sancti-felicis*	Apiol (76.1%)
*P. soledadense*	Limonene (38.5%), caryophyllene oxide (13.3%), γ-muurolene (5.8%)
*P. tuberculatum*	β-Bisabolene (40.2%), δ-cadinene (9.8%), β-caryophyllene (9.7%), germacrene D (5%)

**Table 2:** Antifeedant effects of the essential oils from *Piper* species, the main components of the active oils and myristicin on *S. littoralis*, *M. persicae*, and *R. padi*.

Species/Compounds	%FI-SI ^a^ EC_50_ ^b^	*S. littoralis*	*M. persicae*	*R. padi*
*P. anonifolium*	%FI-SI	30 ± 8	69 ± 5 *	52 ± 8 *
	EC_50_	>50	~50	~50
*P. casapiense*	%FI-SI	12 ± 8	67 ± 7 *	76 ± 5 *
	EC_50_	>50	~50	3.8 (1.6–8.8)
*P. coruscans*	%FI-SI	40 ± 5	74 ± 5 *	34 ± 7
	EC_50_	>50	~50	>50
*P. dumosum*	%FI-SI	58 ± 12 *	32 ± 8	40 ± 8
	EC_50_	~50	>50	>50
*P. mituense*	%FI-SI	91 ± 3 *	91 ± 3 *	92 ± 4 *
	EC_50_	15.6 (8.7–27.8)	23.2 (19.1–28.3)	9.7 (5.6–16.9)
*P. obliquum*	%FI-SI	21 ± 9	58 ± 7 *	89 ± 3 *
	EC_50_	>50	~50	25.6 (17.6–35.4)
*P. reticulatum*	%FI-SI	26 ± 13	90 ± 4 *	62 ± 6 *
	EC_50_	>50	16.2 (9.2–28.5)	~50
*P. sancti-felicis*	%FI-SI	90 ± 4 *	79 ± 5 *	97 ± 1 *
	EC_50_	42.0 (35.3–49.8)	53.5 (40.6–70.5)	8.6 (5.6–13.4)
*P. soledadense*	%FI-SI	48 ± 13	44 ± 9	56 ± 7
	EC_50_	>50	>50	~50
*P. tuberculatum*	%FI-SI	9 ± 6	77 ± 5 *	39 ± 7
	EC_50_	>50	39.5 (29.1–58.0)	>50
Apiol	%FI-SI	81.84 ± 11.00 *	95.32 ± 1.59 *	99.07 ± 0.93 *
	EC_50_	22.24 (16.27–29.73)	16.00 (11.09–21.85)	17.70 (14.79–21.78)
β-Bisabolene	%FI-SI	29.94 ± 16.16	48.18 ± 9.48	75.03 ± 6.94 *
	EC_50_	>50	>50	26.51 (20.79–32.51)
δ-Cadinene	%FI-SI	16.24 ± 11.02	45.20 ± 9.60	53.10 ± 7.46
	EC_50_	>50	>50	>50
β-Caryophyllene	%FI-SI	17.02 ± 10.44	26.7 ± 7.28	46.71 ± 7.78
	EC_50_	>50	>50	>50
β-Caryophyllene oxide	%FI-SI	55.20 ± 12.90 *	24.63 ± 7.66	24.69 ± 7.20
	EC_50_	~50	>50	>50
α-Humulene	%FI-SI	29.99 ± 15.79	46.90 ± 9.36	33.13 ± 7.22
	EC_50_	>50	>50	>50
Limonene	%FI-SI	26.14 ± 8.69	38.39 ± 8.77	31.47 ± 7.12
	EC_50_	>50	>50	>50
Myristicin	%FI-SI	66.93 ± 9.80 *	47.15 ± 9.50	71.16 ± 7.37 *
	EC_50_	~50	>50	25.50 (21.10–29.82)
Nerolidol ^c^	%FI-SI	25.91 ± 16.44	60.94 ± 10.3 *	54.02 ± 8.34 *
	EC_50_	>50	≅50	≅50
β-Pinene	%FI-SI	30.87 ± 7.67	35.04 ± 7.49	34.63 ± 6.27
	EC_50_	>50	>50	>50
α-Pinene	%FI-SI	49.16 ± 16.18	26.61 ± 7.50	23.25 ± 5.74
	EC_50_	>50	>50	>50
Thymol	%FI-SI	85.49 ± 5.05	80.85 ± 7.70	92.07 ± 2.58
	EC_50_	21.0 0(14.5–27.1)	7.60 (4.1–14.4)	18.60 (4.123.3)

^a^ %FI-SI = [1 − (T/C)] × 100, where T and C are the consumption/settling of treated and control leaf disks, respectively. ^b^ Effective antifeedant dose (EC_50_) and 95% confidence (lower, upper) (µg/cm^2^). ^c^ Ref. [31]. * Significantly different from the control, *p* > 0.05, Wilcoxon signed rank test.

**Table 3:** Ixodicidal activity of *Piper* oils, the main components of the active oils, and myristicin against *Hyalomma lusitanicum* larvae.

Essential Oil	Mortality ^a^	LD_50_ [µg/mg Cellulose]	LD_90_ [µg/mg Cellulose]
*P. anonifolium*	35 ± 8	>40	>40
*P. casapiense*	53 ± 7	>40	>40
*P. coruscans*	42 ± 19	>40	>40
*P. dumosum*	100 ± 0	8.891 (8.304–9.600)	13.813 (12,619–15.566)
*P. mituense*	100 ± 0	1.457 (1.323–1.616)	2.171 (1.962–2.469)
*P. obliquum*	38 ± 5	>40	>40
*P. reticulatum*	100 ± 0	6.190 (5.564–6.884)	9.684 (8.733–11.077)
*P. sancti-felicis*	100 ± 0	2.139 (1.899–2.372)	3.543 (3.219–4.010)
*P. soledadense*	100 ± 0	10.055 (9.089–11.077)	16.729 (15.066–19.343)
*P. tuberculatum*	7 ± 2	>40	>40
Apiol	100 ± 0	1.966 (1.798–2.162)	6.321 (5.601–7.671)
δ-Cadinene	0	>20	>20
Caryophyllene	19.4 ± 9.52	>20	>20
β-Caryophyllene oxide	100	4.462 (4.020–5.044)	6.321 (5.601–7.671)
Copaene	0	>20	>20
Limonene	6.8 ± 1.84	>20	>20
Myristicin	100	2.885 (2.613–3.198)	4.452 (4.032–5.037)
Nerolidol	10.97 ± 2.01	>20	>20
α-Pinene	0	>20	>20
β-Pinene	0	>20	>20
Thymol	100	1.47 (1.04–1.77)	3.08 (2.65–3.92)

^a^ Mortality values (mean ± standard error) were corrected according to *Schneider orelli* formula (at a dose of 20 and 10 µg/mg cellulose).

## Data Availability

The original contributions presented in this study are included in the article. Further inquiries can be directed to the corresponding authors.

## Supplementary Materials

The following supporting information can be downloaded at https://www.mdpi.com/article/10.3390/molecules31122177/s1, Table S1: GC-MS analysis (% abundance) of ten Amazonian *Piper* species essential oils. Species abbreviations are: Pa (*P. anonifolium*), Pcs (*P. casapiense*), Pc (*P. coruscans*), Pd (*P. dumosum*), Pm (*P. mituense*), Po (*P. obliquum*), Pr (*P. reticulatum*), Psf (*P. sancti-felicis*), Ps (*P. soledadense*), Pt (*P. tuberculatum*). Tentative identification of compounds without a match (>90%) in the databases has been carried out based on retention time, *m*/*z* and retention index. The Excel file can be accessed at DOI: 10.5281/zenodo.20284642. Table S2: Nematicidal activity of *Piper* oils and apiol against *Meloidogyne javanica* juveniles (J2). Table S3: Molecular docking results for apiol and reference ligands across acetylcholinesterase (AChE) and cytochrome P450 (CYP450) enzymes. Docking scores correspond to the most favorable pose among 50 generated conformations. Interaction types (hydrogen bonding, π–π stacking, hydrophobic, and polar contacts) were assigned based on residue chemical properties and spatial proximity within the binding pocket.
