# Supplementary material for: Apiol-Rich and Caryophyllene-Oxygenated Essential Oils from Amazonian Piper Species as Dual-Action Biopesticides: Broad-Spectrum and Selective Antifeedant
Source: Molecules. 2026 Jun 22;31(12):2177. doi: 10.3390/molecules31122177 (PMC13305312; doi:10.3390/molecules31122177)
Supplement: Supplementary file 1 [file molecules-31-02177-s001.zip › molecules-4357648-supplementary.pdf]

Table S1. GC-MS analysis (% abundance) of ten Amazonian *Piper* species essential oils. Species abbreviations are: Pa (*P. anonifolium*), Pcs (*P. casapiense*), Pc (*P. coruscans*), Pd (*P. dumosum*), Pm (*P. mituense*), Po (*P. obliquum*), Pr (*P. reticulatum*), Psf (*P. sancti-felicis*), Ps (*P. soledadense*), Pt (*P. tuberculatum*). Tentative identification of compounds without match (>90%) in the databases have been carried out based on retention time, m/z and retention index. Excel file can be access at DOI: 10.5281/zenodo.20284642.

Table S2. Nematicidal activity of *Piper* oils and apiol against *Meloidogyne javanica* juveniles (J2)

**Essential oil /compound**	**% J2 Mortality**[a]
P. anonifolium	0
P. casapiense	2.14 ± 0.60
P. coruscans	4.41 ± 1.94
P. dumosum	3.54 ± 0.73
P. mituense	7.16 ± 1.92
P. obliquum	0
P. reticulatum	1.02 ± 0.94
P. sancti-felicis	23.97 ± 2
P. soledadense	58.23 ± 8.55
P. tuberculatum	3.28 ± 1.34
Apiol	2.58 ± 0.71
Thymol[1]	100 ± 0.00

[a] Values are means of four replicates corrected according to Scheider-Orelli's formula. Tested at 1mg/mL.

[1]Tested at 0.5 mg/mL

Table S3. Molecular docking results for apiol and reference ligands across acetylcholinesterase (AChE) and cytochrome P450 (CYP450) enzymes. Docking scores correspond to the most favorable pose among 50 generated conformations. Interaction types (hydrogen bonding, π–π stacking, hydrophobic, and polar contacts) were assigned based on residue chemical properties and spatial proximity within the binding pocket.

Species	Protein	Active Site	Ligand	Score	Key Residues	Predicted Interaction Types
Spodoptera littoralis	AChE	Ser313, Glu439, His553	Acetylcholine	−5.457	Trp198, Tyr442, Phe443	π–π stacking, hydrophobic
					Ser313, Glu312	H-bond, polar
			Apiol	−6.038	Trp198, Tyr235, Phe443	π–π stacking, hydrophobic
					Ser313, Glu312	H-bond
					Gly233	hydrophobic/van der Waals
Myzus persicae	AChE	Ser276, Glu407, His521	Acetylcholine	−6.539	Trp145, Phe368, Tyr408	π–π stacking
					Ser276	H-bond
			Apiol	−5.759	Trp145, Phe368, Tyr408	π–π stacking
					Ser276, Glu275	H-bond, polar
					Leu366	hydrophobic
Ixodes scapularis	AChE	Ser222, Glu351, His464	Acetylcholine	−3.829	Phe145, Tyr358, Tyr359	π–π stacking
					Ser222	H-bond
			Apiol	−5.61	Phe145, Tyr358, Tyr359	π–π stacking
					Ser222, Gln221	H-bond, polar
					Gly141, Ala142	hydrophobic
Meloidogyne javanica	AChE	Ser302, Glu361, His482	Acetylcholine	−5.995	Trp188, Tyr223, Tyr364	π–π stacking
					Ser302, Glu329	H-bond
			Apiol	−5.405	Trp188, Tyr223, Phe404	π–π stacking
					Glu361	H-bond
					Leu442, Gly224	hydrophobic

Hyalomma asiaticum	CYP3A8	Arg103, Arg104, Leu106	Terpinolene	−5.983	Phe108, Phe236, Phe241	π–π / hydrophobic
					Leu113, Met116	hydrophobic
			Apiol	−5.345	Phe108, Phe236, Phe241	π–π stacking
					Arg103, Arg104	polar / electrostatic
					Ser118	H-bond
Drosophila melanogaster	CYP6A2	Arg103, Phe109, Ile119	α-Pinene	−5.006	Phe228, His117	π–π / hydrophobic
					Asn120	H-bond
			Apiol	−5.832	Phe228, Phe305	π–π stacking
					Asn120	H-bond
					Val306, Val309	hydrophobic
